# Success of an Inpatient Rehabilitation Program in Subjects with Type 2 Diabetes Mellitus with or Without Metabolic Syndrome

**DOI:** 10.3390/biom14121527

**Published:** 2024-11-28

**Authors:** Bettina Thauerer, Anna Odrovicsné Tóth, Andreas Salzer, Bibiane Steinecker-Frohnwieser

**Affiliations:** 1 Ludwig Boltzmann Institute for Arthritis and Rehabilitation, 5760 Saalfelden, Austria; anna.odrovicsne-toth@ar.lbg.ac.at; 2 Rehabilitation Center Saalfelden of the Pension Insurance Institution, 5760 Saalfelden, Austria; andreas.salzer@pv.at; 3 Ludwig Boltzmann Institute for Arthritis and Rehabilitation, 8962 Gröbming, Austria

**Keywords:** type 2 diabetes mellitus (T2DM), metabolic syndrome (MetS), advanced glycation end products (AGEs), soluble receptor for AGE (sRAGE), lipid metabolism, oxidative stress, prediction model, rehabilitation outcome

## Abstract

Introduction: Type 2 diabetes mellitus (T2DM) comprises heterogeneous disorders, which have an increase in blood glucose concentrations in common. Metabolic syndrome (MetS) describes the simultaneous occurrence of several clinical symptoms that increase the risk of cardiovascular disease and T2DM, although T2DM itself is also considered a risk factor for developing MetS. Objective: This study aimed to identify parameters related to rehabilitation success and relevant to MetS in T2DM patients. Methods: T2DM patients were divided into two subgroups based on the NHLBI/AHA and IDF guidelines for characterizing MetS. Serum samples were analyzed for T2DM-specific parameters, lipid metabolism, oxidative processes, AGE activity (AAct), and uric acid to HDL ratio (UHR) at admission and discharge after a 3-week inpatient rehabilitation stay. Logistic regression and before–after comparisons were performed showing the importance of multidisciplinary rehabilitation. Results: Among eighty-six patients, 59.3% had MetS. Significant differences between subgroups were found in fasting glucose (FBS), hemoglobin A1c (HbA1c), high-density lipoprotein cholesterol (HDL), triglycerides (TGLs), soluble receptor for AGE (sRAGE), UHR, and AAct. Rehabilitation-induced changes in disease-related parameters were influenced by the presence of MetS. The predictive capacity from all parameters together could be reduced within the three weeks. Conclusion: Rehabilitative measures have a major influence on MetS-relevant factors and can change the course of the disease in patients with T2DM. Identifying these factors can be of great importance for future diagnoses and treatments of T2DM and MetS.

## 1. Introduction

The chronic metabolic disease diabetes mellitus is a rapidly growing global problem with enormous social, health, and economic consequences [1]. According to the *IDF Diabetes Atlas 2021*, more than 10% of adults (aged 20 to 79) worldwide have diabetes, and projections suggest that the number of these patients could reach 783 million (12% of the world’s population) by 2045 [2]. Moreover, the incidence of T2DM has increased dramatically in all countries over the past 3 decades [3]. An estimated 20–25% of the world’s adult population has MetS, and the risk of developing T2DM is five times higher [4].

A high caloric intake by today’s dietary standards and a physically low-active lifestyle have caused a sharp increase in obesity, significantly increasing the risk of developing T2DM and MetS. Metabolic syndrome (MetS) and T2DM are closely linked. Metabolic syndrome is a cluster of risk factors that include abdominal obesity, insulin resistance, high blood pressure, high cholesterol, and elevated blood sugar levels. While MetS increases the risk of T2DM, the presence of diabetes alongside MetS can exacerbate the progression and severity of both conditions. When diabetes patients also have metabolic syndrome, their risk for severe complications increases significantly. Both conditions increase the likelihood of developing cardiovascular diseases, kidney problems, nerve damage (neuropathy), and eye disease (retinopathy). The combination of insulin resistance, obesity, and other factors accelerates the development of these complications.

MetS itself is described by a clustering of risk factors that significantly increase the likelihood of developing T2DM, cardiovascular diseases, stroke, or all three. Key factors include abdominal obesity, high blood pressure, impaired fasting blood sugar, high TGL, and low HDL. Following the harmonization, the NHLBI/AHA and IDF guidelines for characterizing MetS in 2009, MetS is diagnosed when an individual has three or more of the relevant factors specified therein [5]. Several studies have shown that patients with T2DM have a higher incidence and risk of developing MetS than healthy people [6,7].

Importantly, the main goal of treatment for patients with MetS is to reduce the risk of developing heart diseases and to prevent or reduce the development and worsening of T2DM. The most important elements in the treatment of MetS are lifestyle changes such as a healthy diet, weight loss, regular physical activity, stress management, smoking cessation, and adequate sleep. Also, different types of medication may be required, such as drugs to lower blood pressure, control TGL and HDL levels, or lower blood glucose levels [8]. Uric acid (UA), as a novel biomarker for MetS produced through purine metabolism, has been linked to various conditions such as hypertension, heart failure, coronary artery disease, chronic kidney disease, and diabetes mellitus [9,10,11,12]. Interestingly, Mahajan et al. showed that a higher blood UA level was prevalent in patients with MetS [13] and evidence suggests that the UA to HDL cholesterol ratio (UHR) is elevated in patients with inflammatory conditions. Recently, UHR has emerged as a novel metabolic marker, showing increased levels in conditions like steatohepatitis and Hashimoto’s thyroiditis, and its predictive value has been confirmed for T2DM and MetS [14,15]. Hyperglycemia in T2DM causes non-enzymatic glycation of free amino groups of proteins and leads to their structural and functional changes. This reaction leads to the formation of AGEs, which are also known to be involved in the pathophysiology of diabetic complications [16]. Recent studies suggest that the ratio of AGEs to sRAGE is a more accurate biomarker of AGE-related disease than either alone [17].

Furthermore, low-grade inflammation and increased inflammatory proteins play a key role in T2DM, with C-reactive protein (CRP) as the primary marker [18]. Reduced insulin secretion leads to chronic hyperglycemia, causing microvascular and macrovascular complications, and contributing to higher mortality in T2DM patients [19]. The glycated hemoglobin (HbA1c) as a marker of glycemic control represents the average blood glucose level of the last 2–3 months, thereby playing a crucial role in the diagnosis and monitoring of patients with T2DM [20]. Moreover, people with T2DM or MetS often exhibit dyslipidemia, including hypertriglyceridemia and an increased risk of hypercholesterolemia due to insulin resistance and higher fatty acid influx to the liver [21]. This leads to elevated very low-density lipoprotein, which converts to LDL, increasing CVD risk in T2DM patients [22].

Oxidative stress is a key factor in the pathogenesis and progression of diabetes and its complications. An imbalance between reactive oxygen species (ROS) and antioxidant defenses leads to cellular damage. A low level of total antioxidant capacity (TAC) is linked to the pathogenesis and chronic complications of T2DM, affecting both microvascular and cardiovascular systems [23]. Oxidized LDL (oxLDL), associated with obesity and inflammation, acts as a pro-oxidant and is a predictor of T2DM [24]. Uncontrolled myeloperoxidase (MPO) release worsens inflammation, oxidative stress, and metabolic dysfunction, promoting atherogenesis, with higher serum MPO levels observed in diabetics [25].

The underlying cause of MetS is still a challenge for experts, highlighting the importance of early diagnosis and treatment. This study aimed to assess T2DM patients with and without MetS at the beginning and end of a three-week inpatient rehabilitation program to test for relevant rehabilitation success affecting MetS and relevant associated markers. In addition, the presented methodological approach was used to develop predictive models for the assessment of MetS in T2DM patients.

## 2. Materials and Methods

### 2.1. Study Design

The present study was carried out as a cross-sectional study at the rehabilitation center of the Austrian Pension Insurance Institution (PV) in Saalfelden in the period from October 2018 to August 2021. All the procedures described were in accordance with the ethical standards of the Salzburg Ethics Commission (415-E/2338/4-2018) and the Declaration of Helsinki. The concept was approved by the PV. The study was registered in the German Register for Clinical Studies (DRKS00018930).

In this pre–post study, serum and data of the examined subjects were collected at admission (baseline) and at discharge after three weeks of inpatient rehabilitation. Inclusion criteria were the already previously confirmed diagnosis of T2DM, age between 25 and 75 years, and an inpatient rehabilitation stay. Exclusion criteria were the presence of renal insufficiency from creatinine clearance < 60 mL/min, acute inflammation (CRP cut-off > 1.0 mg/dL), surgery or major trauma < 6 weeks ago, myocardial infarction < 10 weeks, pregnancy/lactation period, and alcohol or drug abuse. The patients were informed verbally and in writing. A declaration of willingness to participate was signed. All data were anonymized and processed in accordance with the data protection guidelines.

### 2.2. Study Subject Recruitment

Ninety-two patients were assessed for eligibility. Two of them were excluded from the study because they did not meet the inclusion criteria, and four dropped out and discontinued the rehabilitation program. Thus, data from 86 patients with confirmed T2DM since 4.4 ± 1.0 years, aged 35–73 years, were used for further analysis (Figure S1). For three-weeks, all participating subjects underwent an inpatient multidisciplinary rehabilitation program combined with active and passive physical therapy, following a medical admission procedure. In addition to physiotherapeutic standards, patients also received medical training therapy (individually adapted strength and endurance training), cycle ergometer training (this is tailored to the patient, where the heart rate measurement is monitored to avoid overloading), diet and nutritional consultations, patient education program, and psychosocial support. The patient is monitored medically by the doctor throughout the entire period.

As physiological biomarker, at the beginning and at the end of the rehabilitation stay, the individual’s physical capacity was monitored using an Ergoselect 200 Ergometer (Ergoline GmbH, Bitz, Germany) by AMEDTEC ECGpro software Vs. 5.10.007, in accordance with the manufacturer’s instructions.

### 2.3. Identification Criteria of Patients with Metabolic Syndrome

Diagnosis of MetS seems to be challenging, and different definitions have been used over the past decade depending on the occurrence of different combined MetS parameters, derived from the International Diabetes Federation (IDF), the American Heart Association (AHA), and the National Heart, Lung, and Blood Institute (NHLBI). The main difference concerns the measure for central obesity. In 2009, these major organizations attempted to unify the criteria to identify three or more of the five factors (systolic blood pressure ≥ 130 mmHg or diastolic blood pressure ≥ 85 mmHg; FBS ≥ 100 mg/dL; waist circumference ≥ 94 cm in men and 80 cm in women, or BMI > 30 kg/m^2^; HDL < 40 mg/dL in men and <50 mg/dL in women; TGL ≥ 150 mg/dL) in a patient that would establish the diagnosis of MetS [5]. Based on this standardized categorization, we selected patients.

### 2.4. Analysis of Serum Samples

Serum blood samples were taken at the beginning and at discharge of rehabilitation stay; they were centrifuged within one hour and stored at −80 °C. The following three parameter panels were measured: (1) T2DM-specific parameters: CRP, FBS, and HbA1c. CRP was determined using particle-enhanced immunological turbidity test (Cat. No. 05336163 190, Roche Diagnostics GmbH, Mannheim, Germany); the FBS concentration was assessed photometrically by UV test (Cat. No. 03039773 190, Roche Diagnostics GmbH, Mannheim, Germany), and the HbA1c determination was based on the turbidimetric immunological inhibition assay for hemolyzed whole blood (Cat. No. 05336163 190; Hemolyzing Reagent, Cat. No. 04528182 190, Roche Diagnostics GmbH, Mannheim, Germany). (2) Lipid metabolism: total cholesterol (Chol), low-density lipoprotein (LDL) cholesterol and HDL, TGL, and UA. Chol concentration was determined using enzymatic and colorimetric methods by measuring the increase in absorbance at 512 nm (Cat. No. 03039773 190, Roche Diagnostics GmbH, Mannheim, Germany); the LDL and HDL determinations were performed by direct homogenous enzymatic color test (Cat. No. 07005717 190, 07528566 190, Roche Diagnostics GmbH, Mannheim, Germany), and the concentrations of TGL and UA were measured photometrically using enzymatic color test (Cat. No. 20767107 322, 03183807 190, Roche Diagnostics GmbH, Mannheim, Germany). The concentration of HbA1c was determined from EDTA blood, while the other parameters were obtained from blood serum. All markers were measured using a Cobas 6000 analyzer (Roche Diagnostics GmbH, Mannheim, Germany). (3) Oxidative process parameters, additionally measured via commercial enzyme-linked immunosorbent assays (ELISAs): oxLDL (Cat. No. 10-1143-01, Mercodia AB, Uppsala, Sweden), MPO (Cat. No. LF-EK0134, Abfrontier Hölzel Diagnostika, Cologne, Germany), TAC (ImAnOx TAS/TAC, Cat. No. K5200, Immundiagnostik AG, Bensheim, Germany), AGEs (Cat. No. MBS267540, MyBioSource, San Diego, CA, USA), and its protective soluble receptor sRAGE (Cat. No. RD191116200R, BioVendor, Brno, Czech Republic). The final detection was performed with an absorbance microplate reader (Infinite F50 from Tecan, Grödig, Austria) at specific wavelengths in duplicates. For quality reasons, the controls included in the kits were used. An intra-assay coefficient of variance (CV) < 10% and an inter-assay CV < 15% were used as an approval criterion. All CV values were within the aforementioned limits.

### 2.5. Calculation of UA to HDL Ratio and AGE Activity

A combination of several parameters is often more precise and reliable than using them individually. For example, the UA to HDL ratio (UHR) is a strong marker for metabolic disorders and can also be used to screen individuals at risk of MetS [26]). Recent studies point to another important biomarker ratio (AGE/sRAGE) for different types of diseases, the so-called AGE activity (AAct). Both AGE and sRAGE play a critical role in T2DM-related processes; however, a number of studies revealed controversial results for either AGE or sRAGE alone. A potential solution to this controversy is to consider the AGE and sRAGE simultaneously and build the ratio of AGE to sRAGE, which might be the most appropriate biomarker for AGE–RAGE axis [27]. Based on these data, we also measured these ratios (UHR and AAct) in our patient population to determine the success of the rehabilitation program.

### 2.6. The Rehabilitation Regiment

The performed rehabilitation program was based on a multimodal therapy concept with a focus on the implementation of agreed participation goals, activity goals, and functional goals, taking their performance into account. Rehabilitation goals were described in terms of the ICF models. To support lifestyle changes, patients received training in case and care management, diabetes and nutritional counseling, advice on heart failure and, if necessary, wound management. These training courses covered 10–30% of the rehabilitation stay. The remaining therapy portion was filled with active services that covered 70–90% such as sports and exercise therapy, physiotherapy, occupational therapy, and other functional therapies. Active therapies usually consisted of strength training, endurance training, such as Nordic walking, but also swimming, ergotherapy, and therapeutic gymnastics. Net therapy minutes amounted 1800 to 2400 min depending on the capacity of the patient.

A high-fiber, mixed diet with improved fatty acid balance that limited cholesterol-rich foods, avoided fast-absorbing carbohydrates, and focusing on whole foods was advised. A fiber-rich, carbohydrate-balanced diet with high-quality fats and proteins (both animal- and plant-based) was emphasized. An adequate intake of vitamins and minerals was ensured through the daily consumption of vegetables, fruit and salads.. Herbs and spices to reduce salt intake and healthy drinks to maintain proper hydration were integrated into the diet. Aiming for 1500 kcal daily, this diet supports blood sugar control and overall health.

### 2.7. Statistical Analysis

Continuous variables are shown as mean ± standard error of mean (SEM). The statistical analysis of the data was performed using the statistic program GraphPad Prism 10.1.2. A *p* < 0.05 was considered statistically significant. Pairwise deletion was used for completely randomly distributed missing data. Wilcoxon matched-pairs signed-rank test or Mann–Whitney test was used for comparison of pre–post groups and between groups, respectively. Correlation analyses were carried out using Spearman’s rank correlation coefficient (r). Univariate and multivariate logistic regression analyses were applied to screen the indicators with risk value. Receiver operating characteristics (ROCs) were used to assess the prediction efficiency. It was assumed that an AUC (area under the curve) ≥ 0.5 has predictive features and that the assessment efficiency increases in line with the increase in area.

## 3. Results

### 3.1. Characteristics of Patients

During the study period, 86 patients underwent a three-week inpatient rehabilitation. The cohort with a mean age of 60.0 ± 0.84 years comprised 73.26% male patients. All included persons had had T2DM for 4.4 ± 1.0 years, and the prevalence of metabolic syndrome was 59.30%. The prescribed medications contained the following main groups of active ingredients: metformin (81.69%), gliflozin (35.21%), DPP-4 inhibitors (21.13%), glutide (7.04%), and sulfonylurea (4.23%), and 38.03% of the patients used medications with more than one active ingredient. A total of 14.08% of the patients required insulin. The examined subjects showed a body mass index (BMI) of 30.69 ± 0.55 kg/m^2^ and a value of 123.80 ± 5.05 Watt for the bicycle ergometer training. The waist circumference was measured in 31 patients within the cohort. This amounted to 111.17 cm at the beginning and 108.3 on discharge.

### 3.2. Characterization of the Patient Groups with/Without Metabolic Syndrome

The patients included in this study were divided into two subgroups according to the 2009 harmonized criteria: with (MetS(+)) or without MetS (MetS(−)). The baseline characteristics of patients with T2DM were compared between these two subgroups (Table 1). FBS was 27.2% and HbA1c was 14.4% higher in the MetS(+) than in the MetS(−) subgroup. By comparing the parameters involved in lipid metabolism, significant differences between the subgroups could be detected with a 24.9% reduction in HDL and a 89.9% increased TGL level in the MetS(+) group when compared to MetS. Except for sRAGE, with a reduction by 31.0% in the MetS(+) subgroup, investigated oxidative processes parameters did not show altered concentrations across the subgroups. Values at admission to rehabilitation for the specific ratio parameters such as the UHR and the AAct were also assessed. In the MetS(+) subgroup, a significant increase of 33.1% for UHR and of 23.1% for AAct compared to MetS(−) could be detected.

### 3.3. Identification of Parameters Predicting the Occurrence of the Metabolic Syndrome

**Figure 1:**
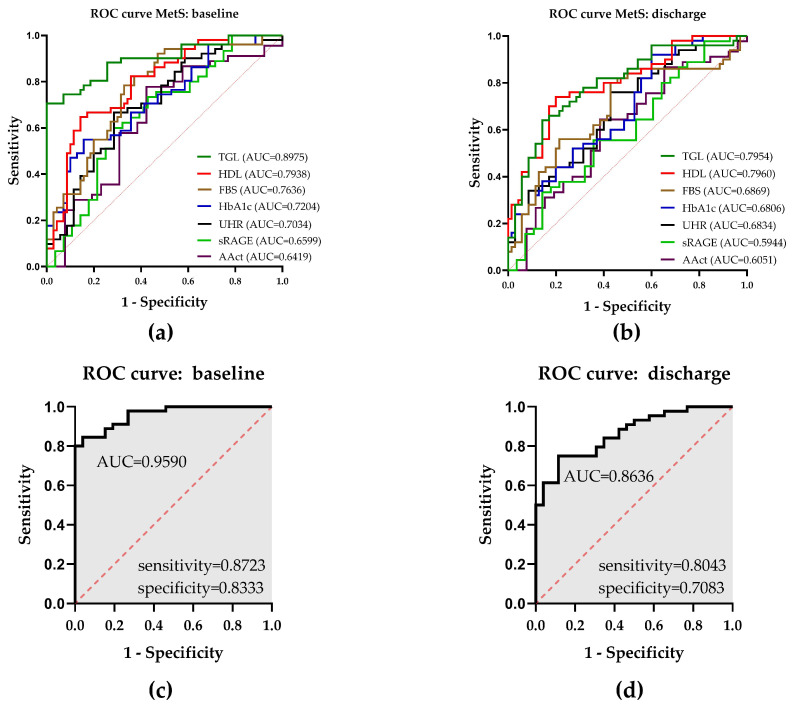
Univariate and multivariate logistic regressions for the appearance of MetS. (**a**) ROC for chosen parameters with subgroup differences at admission to rehabilitation and (**b**) at discharge. Multivariate logistic regression with TGL, HDL, FBS, HbA1c, UHR, sRAGE, and AAct (**c**) at admission and (**d**) discharge. AUC, sensitivity, and specificity are shown within the graphs. The red dashed line represents the chance discrimination level.

An ROC curve analysis was performed with parameters which showed significant differences between the two subgroups to determine which could predict the possible incidence of MetS. Univariate logistic regression analysis of parameters at admission (Figure 1a) revealed that TGL had the best predictive characteristic by showing an area under the curve (AUC) = 0.8975 followed by HDL (AUC = 0.7938), FBS (AUC = 0.7636), HbA1c (AUC = 0.7204), and UHR (AUC = 0.7034). sRAGE (AUC = 0.6599) and AAct (AUC = 0.6419) displayed only low predictive functions with regard to the presence of MetS. A reduction in predictive characteristics (AUC) of all tested parameters could be seen by using samples from the discharge for analysis (TGL = 0.7954, HDL = 0.7960, FBS = 0.6869, HbA1c = 0.6806, UHR = 0.6834, sRAGE = 0.5944, and AAct = 0.6051) (Figure 1b). Subsequent multivariate regression analysis at baseline (all parameters from univariate regression analysis) revealed a high predictive function (AUC = 0.9590) with a positive predictive power of 87.23% (sensitivity) and a negative predictive power of 83.33% (specificity) (Figure 1c). A multivariate regression analysis, performed with blood samples taken after three weeks rehabilitation showed diminished prediction characteristics (AUC = 0.8636, 80.43% sensitivity, and 70.83% specificity) compared to admission (Figure 1d).

### 3.4. Determining Correlations Between Baseline and Outcomes of Rehabilitation

**Figure 2:**
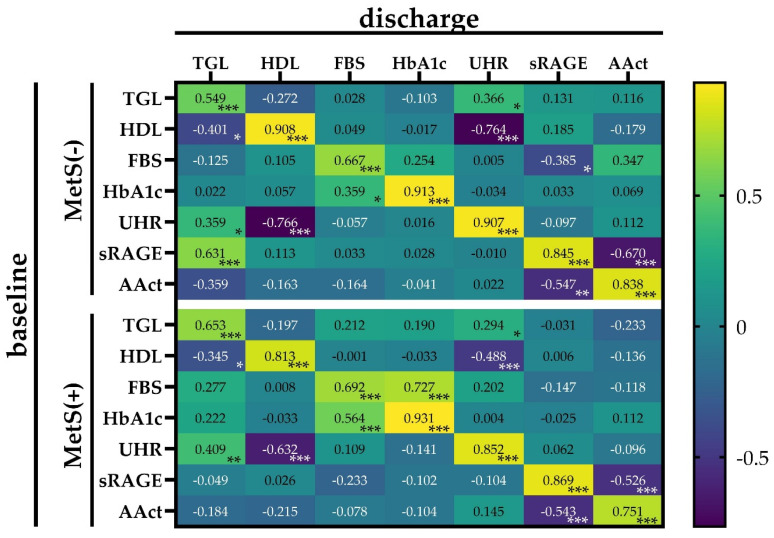
Heatmap reporting Spearman correlation coefficient (r) and significances referred to correlations between baseline and discharge values of the predictive parameters in the MetS(−) and MetS(+) subgroups. The bar on the right side of the map indicates the color legend of the Spearman correlation coefficients, and the asterisk within the squares indicates the significances for each pair of parameters in the matrix. *: *p* < 0.05; **: *p* < 0.01; ***: *p* < 0.001.

In order to clarify how and if the discharge values are related to the baseline values examined, correlation analyses were performed for parameters which were identified as predictive by ROC analysis in this study. The heat plot (Figure 2) for the subgroups MetS(−) and MetS(+) showed differences in the correlation matrix. While TGL_baseline_ showed positive correlations with sRAGE_discharge_ (r = 0.131) and AAct (r = 0.116) in MetS(−) patients, negative links were found in patients with MetS. Differences in the correlation with FBS_baseline_ were found between the subgroups: a positive relation with HbA1c_discharge_ (r = 0.727) was only analyzed in MetS(+) patients, but a negative correlation with sRAGE_discharge_ (r = −0.385) was found only in MetS(−). When comparing the two subgroups, we found sign differences in the correlation between baseline HbA1c and the discharge values of UHR and sRAGE and furthermore between the baseline level of UHR and the discharge values of FBS, HbA1c, sRAGE, and AAct. The strong positive correlation between sRAGE_baseline_ and TGL_discharge_ (r = 0.631), seen in patients without MetS, was not present in the MetS(+) subgroup. It was obvious that in the MetS(−) subgroup, the baseline values of sRAGE were positively correlated with TGL, FBS, and HbA1c, whereas in the other subgroup, the parameters were inversely coupled. The most significant differences between the subgroups were thus observed in the group of T2DM-specific factors and also in sRAGE.

### 3.5. Different Effects of a Three-Week Inpatient Rehabilitation Stay on Patients with or Without Metabolic Syndrome

**Figure 3:**
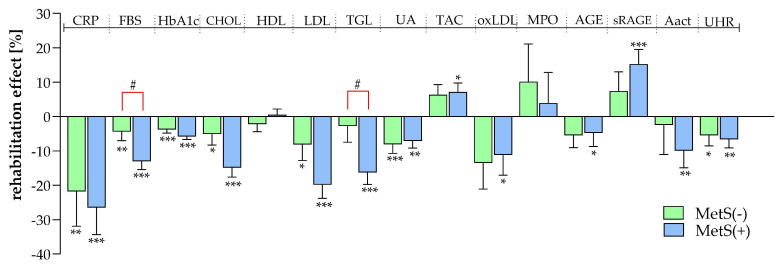
Rehabilitation effect in patients with and without MetS. Comparison between admission and discharge levels given as percentage change (rehabilitation effect %) within each subgroup MetS(−) and MetS(+). Significances were calculated via the Wilcoxon matched-pairs signed-rank test (*: *p* < 0.05; **: *p* < 0.01; ***: *p* < 0.001). For significances between the subgroups, the Mann–Whitney test was used (#: *p* < 0.05).

Up to this point, only the baseline values of the patients have been considered. However, to assess how an inpatient rehabilitation impacts the two subgroups and if they respond differently to rehabilitation, pre–post comparisons and subgroup analyses were conducted (Table S1). Figure 3 represents the percentage change (differences between discharge and baseline in percent; significant differences are indicated by asterisk) for all measured parameters in the MetS(−) and MetS(+) subgroups. An improvement in the measured parameters was shown as a result of the inpatient rehabilitation treatment. Among the T2DM-specific parameters, all factors showed significant reductions between admission and discharge, with the MetS(+) group exhibiting 1.2-fold greater changes in CRP (MetS(−): −21.8% ± 10.1 and MetS(+): −26.5% ± 7.9) and an 3.0-fold higher reduction in FBS (MetS(−): −4.4% ± 2.6 and MetS(+): −13.0% ± 2.4). Even a comparison between the groups showed a significant difference (rhomb). Cholesterol, LDL, and TGL, important parameters in lipid metabolism, showed significant reductions in serum concentrations during the rehabilitation period (Chol: MetS(−): −5.1% ± 3.2 and MetS(+): −14.9% ± 2.8; LDL: MetS(−): −8.2% ± 4.6 and MetS(+): −19.8% ± 3.9; TGL: MetS(−): −2.8% ± 4.7 and MetS(+): −16.3% ± 3.5). The MetS(+) group exhibited significantly greater improvements, with TGL values decreasing 5.8-fold more in this subgroup compared to the MetS(−) subgroup (rhomb). While factors related to oxidative processes showed no significant changes during the rehabilitation period in the patients without MetS, oxLDL, AGE, sRAGE, and AAct exhibited notable alterations in the MetS(+) subgroup. A comparison of the subgroups revealed a 2.1-fold increase (sRAGE) and a 4.1-fold reduction (AAct). The rehabilitation program improved the UHR in both subgroups, whereby the patients with MetS recorded a 1.2-fold greater decrease.

## 4. Discussion

The present study could provide evidence about the favorable effect of a multidisciplinary three-week inpatient rehabilitation program with regard to T2DM and MetS. In the last decades, the prevalence of patients with T2DM and its comorbidity, metabolic syndrome, has increased significantly [7]. It is well established that MetS is linked to a range of disorders and other pathologies that result in significant changes to the human body, particularly impacting patients with T2DM. Making appropriate changes, such as improving diet and achieving weight loss, are essential first steps in the treatment of MetS [28]. Rehabilitation is essential in managing metabolic syndrome and diabetes by fostering lifestyle changes that improve blood sugar control, lower cardiovascular risks, and enhance metabolic health. Programs typically involve exercise, nutrition guidance, behavioral support, and self-management education, helping individuals reduce blood glucose, improve insulin sensitivity, and lower blood pressure. Research shows that participants benefit significantly, experiencing reduced HbA1c, lower cardiovascular risk, weight loss, and improved quality of life. Rehabilitation supports immediate health improvements and empowers individuals with lasting skills for long-term condition management, making it highly effective against complications of metabolic syndrome and diabetes. Knowing that multidisciplinary inpatient rehabilitation encompasses key therapeutic elements for treating this disease, we aimed to compare and analyze T2DM patients with and without MetS at both the beginning and the end of their rehabilitation stay [8]. 

From the outset of our study, when we established the two subgroups and utilized parameters outlined in clinical guidelines to classify patients with MetS, we were able to corroborate the existing literature [29]. Specifically, in our cohort of patients participating in a three-week inpatient rehabilitation program, the incidence of MetS was found to be 59.3%. This finding emphasizes the significant overlap between T2DM and MetS in clinical settings. The relatively high incidence of MetS within our patient population highlights the need for healthcare providers to be vigilant in screening for MetS in T2DM patients, given the associated risks of cardiovascular diseases and other complications. Moreover, it underscores the importance of integrated treatment approaches that address both T2DM and its MetS components to improve patient outcomes during rehabilitation and beyond. Our results contribute to the growing body of evidence advocating for a more comprehensive understanding of the interplay between T2DM and MetS, suggesting that targeted interventions could be pivotal in managing these interconnected conditions effectively. In recent years, MetS has gained considerable attention, prompting various expert groups to establish simple diagnostic criteria that can be easily implemented in clinical practice to identify patients who have multiple components of this condition. While these criteria may vary in their specific elements, they typically include a combination of basic factors and metabolic risk indicators [5]. The goal of this effort is to provide healthcare professionals with practical tools to recognize and effectively treat MetS. In the patient population studied, we were able to clearly demonstrate that the specific parameters recognized internationally for the identification of MetS, namely FBS, HDL, and TGL, play an extremely important role, as significant differences were already found in the subgroups at the start of rehabilitation. Importantly, in addition to these factors, both, elevated HbA1c and reduced sRAGE levels were found in subjects suffering from MetS, which is consistent with previous studies using HbA1c and sRAGE for identification [30,31]. Furthermore, it is well known that the UA is commonly increased in patients with MetS or diabetes mellitus and HDL, in addition to its function in lipid metabolism, plays a crucial role is suppressing the reactions of blood oxidation [32]. 

Furthermore, several earlier studies have indicated that UHR serves as a significant marker for MetS in individuals with T2DM [26]. In line with these findings, our research proved a strong association between a markedly elevated UHR and the presence of MetS in comparison to patients who do not have this condition. 

On the other hand, AGEs play a key role in the pathophysiology of diabetic complications and the interaction between AGEs and their cellular receptor can activate several types of signaling pathways (MAPK/ERK, TGF-β, JNK, and NF-κB) leading to oxidative stress and inflammation [33]. By interaction of AGEs with the decoy receptor sRAGE, the signaling transduction can be interrupted. Using this feature of the AGE axis, new therapeutic interventions have been presented that may act on the AGE/sRAGE signaling pathway and may contribute to slowing the progression of diabetes-related complications [16]. Prior findings revealed that varying diseases exhibit distinct levels of AAct and a three-week inpatient rehabilitation stay positively influences this AGE activity ratio in patients with cardiovascular disease [34]. This observation further emphasizes the potential of AGE activity as a relevant biomarker for the assessment of disease states and associated health-related conditions 

Our current results show that the rehabilitation outcomes of patients with and without MetS differ significantly. Interestingly, patients with MetS demonstrated greater improvements in relevant biomarkers than the T2DM-only group. This suggests that patients with MetS may respond differently to therapy than patients without MetS, emphasizing the importance of tailored rehabilitation strategies. Furthermore, it can also be discussed that patients with MetS had a changed baseline situation at the start of rehabilitation and may therefore represent the more suitable or needier patient cohort. Since MetS is a very significant challenge for global public health, it is important to predict the occurrence of MetS and the associated risk factors. Results of different research groups have previously shown that different parameters and their combinations can be used to predict MetS. 

In our study, we examined parameters that showed significant differences between the MetS(−) and MetS(+) subgroups and identified TGL, HDL, FBS, HbA1c, UHR, sRAGE, and AAct as potential factors for the analysis in creating a predictive model. Although each of these markers is already a strong individual predictor, the combined efficacy of all seven markers could not be surpassed. We investigated how parameters associated with metabolic syndrome can be specifically modified through a three-week multidisciplinary inpatient rehabilitation program to positively influence disease progression, severity, and development of comorbidities. Our findings show that such a program can significantly reduce the risk parameters for predicting the onset of metabolic syndrome in patients with T2DM within only three weeks, thereby weakening the predictive power of these markers. Research by O’Neill showed that factors such as obesity, elevated blood pressure, and low HDL levels could serve as predictors of the risk of developing MetS [35]. In addition, Yuging Li’s results showed that the predictive power of TGL-related parameters was stronger in both men and women. Another study suggests that FBS is more closely associated with MetS and cardiovascular risk biomarkers than HbA1c [36]. Consistent with these findings, our univariate logistic regression analysis showed that FBS had the third highest predictive value among the parameters we examined. Additionally, it is noteworthy that TGL alone can predict MetS in patients with diabetes with quite high accuracy, further suggesting that the prediction of MetS may depend on the underlying condition. Our correlation analyses have provided a more comprehensive view of if and how parameters with predictive characteristics are related to rehabilitation outcomes. Considering T2DM-specific parameters, we also observed significant differences in sRAGE between the two groups of patients studied. In addition to these markers, it is very important that TGL as a basic parameter of lipid metabolism and UHR are also well monitored during rehabilitation. It was clearly demonstrated that the elevated serum CRP concentration is associated with higher fasting insulin and glucose and HbA1c levels, suggesting a possible role of inflammation in processes closely related to the MetS such as insulin resistance and glucose intolerance. The positive correlations observed in TGL are supported by the results of several previous studies showing a strong correlation between lipid metabolism and the pathogenesis of insulin resistance and the development of MetS [37]. 

Our findings align with previous research indicating that serum levels of sRAGE are inversely correlated with various metabolic parameters and higher concentrations of sRAGE are linked to a lower prevalence of MetS [28]. As we can deduce from our results, a three-week rehabilitation stay leads to an increase in serum levels of sRAGE, with this increase being particularly pronounced in patients with MetS. This might also indicate that sRAGE may play a critical role in rehabilitation outcomes and should be considered for future studies.

## 5. Conclusions

Taken together, our results strongly demonstrate that a three-week multidisciplinary inpatient rehabilitation stay has a positive effect on T2DM-specific parameters, namely, UHR, TGL, and serum levels of sRAGE, in subjects suffering from T2DM and MetS. Furthermore, we can conclude that the predictive factors associated with the development of MetS are extremely important for the diagnosis and treatment of the disease.

## Figures and Tables

**Table 1:** Baseline values of parameter panels in MetS subgroups (*: *p* < 0.05; **: *p* < 0.01; ***: *p* < 0.001; ns = not significant).

	MetS(−)		MetS(+)		
	Baseline	n	Baseline	n	*p* Value
**(1) Biochemical parameters of T2DM**					
**CRP** (mg/dL)	0.17 ± 0.02	33	0.27 ± 0.03	48	ns
**FBS** (mg/dL)	117.74 ± 3.69	34	149.72 ± 5.79	50	***
**HbA1c** (mmol/mol)	46.11 ± 1.10	35	52.76 ± 1.37	49	***
**(2) Biochemical parameters of lipid metabolism**					
**Chol** (mg/dL)	151.60 ± 7.62	35	153.60 ± 6.98	50	ns
**HDL** (mg/dL)	52.44 ± 2.28	34	39.38 ± 1.36	50	***
**LDL** (mg/dL)	84.06 ± 6.72	32	86.22 ± 5.76	49	ns
**TGL** (mg/dL)	107.72 ± 4.30	32	204.52 ± 12.78	50	***
**UA** (mg/dL)	5.84 ± 0.25	35	5.93 ± 0.22	50	ns
**UHR**	0.121 ± 0.008	34	0.161 ± 0.010	50	**
**(3) Parameters involved in oxidative processes**					
**TAC** (µmol/L)	297.52 ± 10.40	35	304.20 ± 8.00	51	ns
**oxLDL** (U/L)	47.29 ± 6.00	17	58.88 ± 5.61	31	ns
**MPO** (ng/mL)	210.52 ± 32.83	22	239.00 ± 23.94	43	ns
**AGE** (µg/mL)	9.25 ± 0.95	32	9.23 ± 0.47	49	ns
**sRAGE** (pg/mL)	948.84 ± 128.70	28	654.39 ± 41.90	45	*
**AAct**	0.013 ± 0.002	26	0.016 ± 0.001	45	*

## Data Availability

Restrictions apply to the availability of these data. Data were obtained from the Austrian Pension Insurance Institution (PV) and are available with the permission of PV.

## Supplementary Materials

The following supporting information can be downloaded at: https://www.mdpi.com/article/10.3390/biom14121527/s1. Figure S1: Consort Diagram. Table S1: Baseline and discharge values of parameter panels in MetS subgroups.
