# Supplementary material for: Success of an Inpatient Rehabilitation Program in Subjects with Type 2 Diabetes Mellitus with or Without Metabolic Syndrome
Source: Biomolecules. 2024 Nov 28;14(12):1527. doi: 10.3390/biom14121527 (PMC11673746; doi:10.3390/biom14121527)
Supplement: Supplementary file 1 [file biomolecules-14-01527-s001.zip › Table S1_23102024.pdf]

Table S1. *Baseline and discharge values of parameter panels in MetS subgroups.*

	MetS(-)				MetS(+)			
	**Baseline**	**Discharge**	***p* value**	**n**	**Baseline**	**Discharge**	***p* value**	**n**
**1) Biochemical parameters of T2DM**								
**CRP** (mg/dl)	0.17 ± 0.02	0.12 ± 0.02	**	33	0.27 ± 0.03	0.18 ± 0.03	***	48
**FBS** (mg/dl)	117.74 ± 3.69	110.91 ± 3.02	**	34	149.72 ± 5.79	126.56 ± 4.06	***	50
**HbA1c** (mmol/mol)	46.11 ± 1.10	44.37 ± 1.16	***	35	52.76 ± 1.37	49.49 ± 1.21	***	49
**2) Biochemical parameters of lipid metabolism**								
**Chol** (mg/dl)	151.60 ± 7.62	142.06 ± 8.30	*	35	153.60 ± 6.98	125.42 ± 5.11	***	50
**HDL** (mg/dl)	52.44 ± 2.28	50.50 ± 2.08	ns	34	39.38 ± 1.36	39.16 ± 1.22	ns	50
**LDL** (mg/dl)	84.06 ± 6.72	73.47 ± 6.35	*	32	86.22 ± 5.76	65.00 ± 4.73	***	49
**TGL** (mg/dl)	107.72 ± 4.30	101.78 ± 4.25	ns	32	204.52 ± 12.78	164.40 ± 11.09	***	50
**UA** (mg/dl)	5.84 ± 0.25	5.30 ± 0.23	***	35	5.93 ± 0.22	5.46 ± 0.21	**	50
**UHR**	0.121 ± 0.008	0.113 ± 0.007	*	34	0.161 ± 0.010	0.149 ± 0.009	**	50
**3) Parameters involved in oxidative processes**								
**TAC** (µmol/l)	297.52 ± 10.40	310.37 ± 9.01	ns	35	304.20 ± 8.00	319.27 ± 6.54	*	51
**oxLDL** (U/l)	47.29 ± 6.00	40.61 ± 5.42	ns	17	58.88 ± 5.61	47.56 ± 4.08	*	31
**MPO** (ng/ml)	210.52 ± 32.83	206.66 ± 23.21	ns	22	239.00 ± 23.94	224.74 ± 22.57	ns	43
**AGE** (µg/ml)	9.25 ± 0.95	8.41 ± 0.76	ns	32	9.23 ± 0.47	8.58 ± 0.50	*	49
**sRAGE** (pg/ml)	948.84 ± 128.70	1008.55 ± 182.10	ns	28	654.39 ± 41.90	735.27 ± 46.66	***	45
**AAct**	0.013 ± 0.002	0.012 ± 0.002	ns	26	0.016 ± 0.001	0.013 ± 0.001	**	45
