# Supplementary figures and images for: Success of an Inpatient Rehabilitation Program in Subjects with Type 2 Diabetes Mellitus with or Without Metabolic Syndrome
Source: Biomolecules. 2024 Nov 28;14(12):1527. doi: 10.3390/biom14121527 (PMC11673746; doi:10.3390/biom14121527)

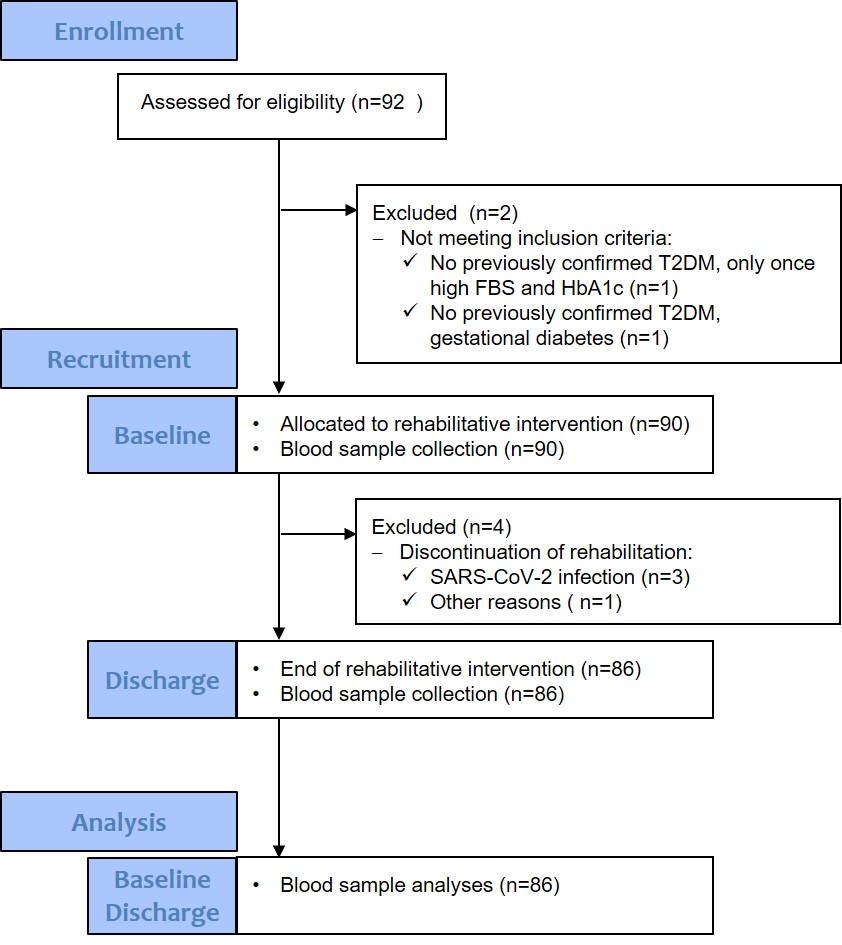

Supplement: Supplementary file 1 [file biomolecules-14-01527-s001.zip › Figure S1 06112024.jpg]
